# Arrhythmogenic Remodeling in the Failing Heart

**DOI:** 10.3390/cells10113203

**Published:** 2021-11-17

**Authors:** Zoltán Husti, András Varró, István Baczkó

**Affiliations:** 1 Department of Pharmacology and Pharmacotherapy, University of Szeged, 6720 Szeged, Hungary; husti.zoltan@med.u-szeged.hu (Z.H.); varro.andras@med.u-szeged.hu (A.V.); 2 Department of Pharmacology and Pharmacotherapy, Interdisciplinary Excellence Centre, University of Szeged, 6720 Szeged, Hungary; 3 ELKH-SZTE Research Group for Cardiovascular Pharmacology, Eötvös Loránd Research Network, 6720 Szeged, Hungary

**Keywords:** arrhythmias, atrial fibrillation, calcium handling, heart failure, remodeling, potassium currents, sudden cardiac death, ventricular fibrillation

## Abstract

Chronic heart failure is a clinical syndrome with multiple etiologies, associated with significant morbidity and mortality. Cardiac arrhythmias, including ventricular tachyarrhythmias and atrial fibrillation, are common in heart failure. A number of cardiac diseases including heart failure alter the expression and regulation of ion channels and transporters leading to arrhythmogenic electrical remodeling. Myocardial hypertrophy, fibrosis and scar formation are key elements of arrhythmogenic structural remodeling in heart failure. In this article, the mechanisms responsible for increased arrhythmia susceptibility as well as the underlying changes in ion channel, transporter expression and function as well as alterations in calcium handling in heart failure are discussed. Understanding the mechanisms of arrhythmogenic remodeling is key to improving arrhythmia management and the prevention of sudden cardiac death in patients with heart failure.

## 1. Introduction

Heart failure is a clinical syndrome with multiple etiologies leading to cardiac function impairment and it is associated with significant morbidity and mortality. According to a recent meta-analysis, the 5- and 10-year survival rates of heart failure are 57%, and 35%, respectively [1]. Previously, up to 50% of sudden deaths in patients with heart failure were attributed to malignant cardiac arrhythmias [2]. Although the incidence of sudden cardiac death (SCD) has been significantly reduced in the last two decades due to advanced medication and the application of implantable ventricular cardioverter/defibrillator devices [3], malignant arrhythmias remain an important cause of SCD in HF patients [4]. In a recent analysis of 12 clinical trials (1995–2014), SCD occurred in 8.9% of patients with HF with reduced ejection fraction (HFrEF; EF ≤ 40%) [5]. Although patients with HF with preserved ejection fraction (HFpEF; EF ≥ 50%) were thought to be at low risk for sudden cardiac death for a long time, recent studies have shown a considerably increased risk. In HFpEF patients in the Irbesartan in Patients with Heart Failure and Preserved Ejection Fraction (I-PRESERVE) trial and the Aldosterone Antagonist Therapy for Adults With Heart Failure and Preserved Systolic Function (TOPCAT) trial, 20% to 26% of the deaths were classified as arrhythmic SCD [6,7]. In addition to severe ventricular arrhythmias, atrial fibrillation (AF) is common in HF [8] and sinus bradycardia can also occur leading to reduced cardiac function [9,10].

A number of pathological settings were shown to cause adaptive and maladaptive changes in the structure, metabolic and electrophysiological function of the heart, and these changes are collectively referred to as remodeling. Some aspects of the structural (myocardial hypertrophy, fibrosis) and electrophysiological (altered ion channel, transporter densities and regulation) remodeling represent a cardiovascular adaptation to the pathophysiological setting, however, these changes may also lead to impaired impulse generation and conduction as well as significant alterations in repolarization and repolarization reserve making the heart predisposed to the generation of arrhythmias.

In this review, the mechanisms responsible for increased arrhythmia propensity and the development of ventricular arrhythmias in heart failure are discussed. In particular, the elements of arrhythmogenic electrical remodeling, including ventricular and atrial action potential, impulse conduction and arrhythmogenic calcium homeostasis alterations are discussed.

## 2. The Proposed Electrophysiological Mechanism of Arrhythmias Leading to Sudden Cardiac Death in HF

In physiological conditions, conduction in the heart is fast (1–2 m/s) and action potential durations in myocardial cells are long (200–300 ms). Since myocardial cells are in a refractory state, they cannot be stimulated early. The effective refractory period (ERP) characterizes the duration of this refractoriness. It should be emphasized that the differences between the action potential durations and therefore the ERPs of neighboring cells are very small in the normal heart resulting in homogenous repolarization. Fast homogeneous conduction and repolarization (and ERP) together prevent circular re-entry excitation and arrhythmias will not develop.

**Figure 1:**
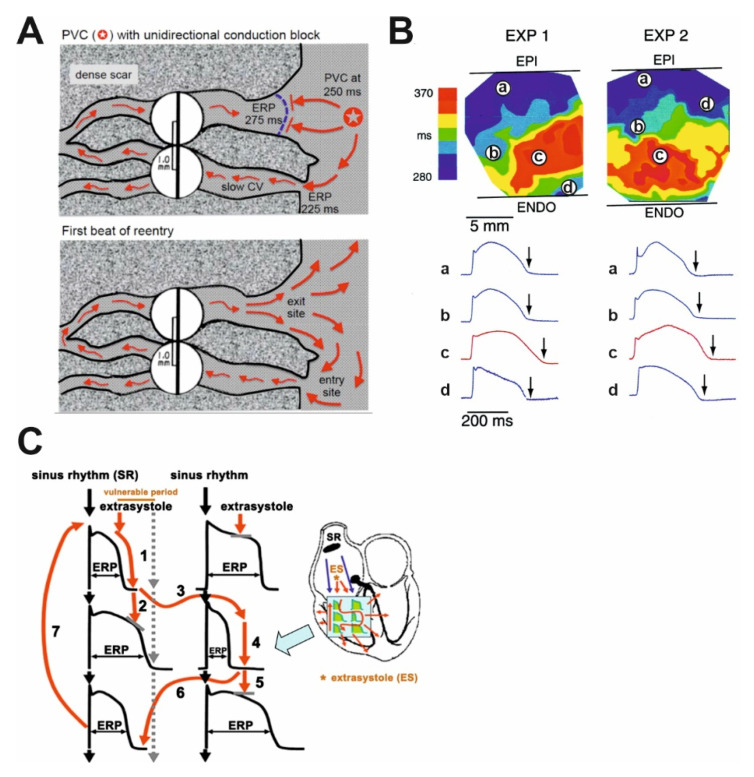
The simplified illustrations of mechanisms creating an arrhythmia substrate for reentry. (**A**) Illustrative example of a classic mechanism by which a premature ventricular complex (PVC) initiates reentry in the fibrotic border zone of the myocardium, due to slow conduction and dispersion of refractoriness. Top panel: a PVC occurring 250 ms following the previous beat arrives too early to conduct through the upper myocyte strand with a long ERP of 275 ms, but it propagates successfully (red arrows) through the lower strand with a shorter ERP of 225 ms (“entry site”). The impulse propagates slowly (slow CV), eventually reaching the upper strand from the opposite direction. Bottom panel: in case the conduction time is >275 ms, the interface of the upper strand with normal tissue (“exit site”) has recovered excitability, and the impulse can then propagate through the region of prior conduction block, thus initiating reentry. Dispersion of refractoriness is caused by electrical remodeling. The slow propagation is due to zig-zag conduction through the myocyte strands as well as gap junction remodeling. CV, conduction velocity; ERP, effective refractory period [15]. (**B**) Two representative experiments (EXP 1 and EXP 2) demonstrate spatial differences in duration of ventricular repolarization. Upper panel shows transmural heterogeneity of action potential durations using color codes during bradycardia and d-sotalol administration mimicking decreased repolarization reserve in the canine wedge long QT syndrome 2 model. Optical action potentials are shown from selected transmural sites (a, b, c, d) on lower panel. Arrows mark significant differences in the action potential duration within relatively short transmural distances. EPI, epicardium; ENDO, endocardium [14]. (**C**) The schematic illustration of functional reentry without a well-defined anatomical obstacle. The arrhythmia substrate is represented by artificially enhanced action potential duration differences. In normal settings, impulses from the sinus node (black arrows) use physiological pathways to propagate through atrial and ventricular tissue and the conduction system. An early trigger (red arrow) can only conduct via pathways where the tissue is not depolarized, and its refractoriness is over, whereas the conduction is blocked in other directions where the tissue is not fully repolarized and cells are refractory. Thus, the abnormal impulse can travel in a complicated pathway through reentry paths created by heterogeneous repolarization and conduction. The dispersion of repolarization creates a time window called the vulnerable period, where triggers could elicit the reentry arrhythmia. However, outside this window extra stimuli would only cause relatively harmless extrasystoles. CV, conduction velocity; ES, extrasystole; SR, sinus rhythm [16].

However, when the duration of repolarization and consequently myocardial ERP are prolonged [11,12], the differences in repolarization duration of adjacent cells also become larger, resulting in enhanced spatial repolarization heterogeneity (Figure 1). Consequently, an early extrasystole in diastole occurring following a normal sinus beat can propagate in the direction of cells with shorter action potential duration, however, its propagation will be blocked or slowed down in the direction of cardiomyocytes with longer action potential durations. In addition, impulse conduction can be impaired due to fibrosis and scars (Figure 1A) observed in HF [13]. Therefore, the extra stimulus can follow a complex path back toward the site of its origin and anywhere else where excitability is regained and can generate polymorphic ventricular tachycardia (VT) or ventricular fibrillation (VF). VF leads to SCD without intervention in a few minutes since it does not revert back to sinus rhythm spontaneously in humans. Importantly, two independent factors are needed for the above-described arrhythmia to occur. The environment for an arrhythmia to develop (“arrhythmia substrate”) is created by prolongation and increased dispersion of repolarization (Figure 1B) supplemented by impaired and/or heterogenous impulse conduction. The presence of an arrhythmia substrate alone, however, is not sufficient for arrhythmia induction. In addition to the presence of an arrhythmia substrate, a “trigger” is also needed to initiate the arrhythmia. The trigger can be provided by an extrasystole (ES) in the vulnerable period that can follow the re-entry paths created by heterogeneous repolarization (Figure 1C). The timing of this trigger ES is essential, in case it occurs before the vulnerable period its conduction will be blocked and following the vulnerable period it will not lead to ventricular tachycardia or fibrillation, only manifests as a harmless single ES. Larger repolarization heterogeneity results in longer vulnerability periods (enhanced “substrate”). More frequent extrasystoles result in increased “trigger” activity and increase the probability of serious arrhythmia development. A similar mechanism for Torsades de Pointes arrhythmia generation was demonstrated experimentally in dogs with increased repolarization heterogeneity using triggering external stimulation [14].

## 3. Heart Failure and the Cardiac Action Potential

**Figure 2:**
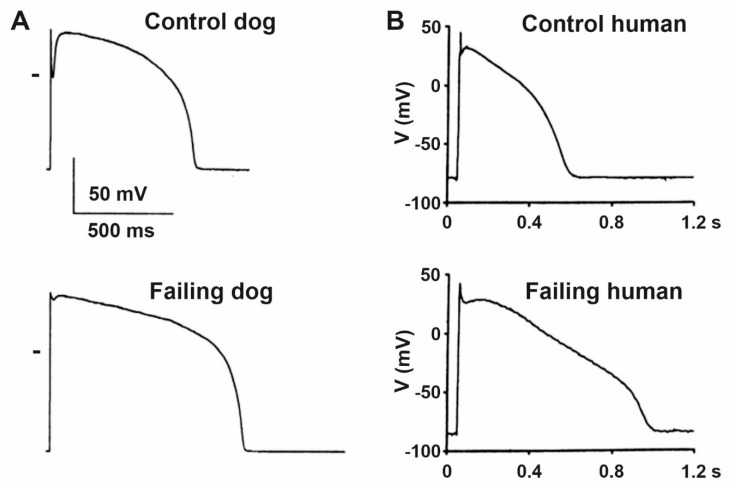
Characteristic ventricular action potentials from control and failing (**A**) dog and (**B**) human ventricular cardiomyocytes show significantly prolonged action potentials with impaired early repolarization notch. Zero voltage is indicated by short bars on panel (**A**) [11,33].

Although the etiologies and manifestations of HF are diverse, the overwhelming majority of reports indicate significant lengthening of cardiac ventricular action potential duration (APD) regardless of the investigated species and its pathophysiological origin in human studies [11,12,17,18] or the experimental techniques applied in different animal models [12,19,20,21,22]. Initially, the prolongation of the ventricular APD may represent an adaptive response to cardiac failure, since it enhances Ca^2+^ entry into the cardiomyocyte resulting in improved contractility [23]. On the other hand, APD prolongation favors early afterdepolarization (EAD) development due to the reactivation of I_CaL_ [24] and I_Na_ [25]. In addition, the lengthening of repolarization (Figure 2) is associated with increased dispersion [18] and enhanced short-term variability of repolarization [26], but it is not necessarily associated with increased transmural repolarization heterogeneity [27]. Action potential prolongation was also reported in Purkinje fibers recorded from failing canine [28] and rabbit [29] hearts. In addition, repolarization alternans (oscillations of long and short action potential durations at rapid heart rates) was also reported in failing heart [30,31] which can be attributed to abnormal function of the calcium handling proteins [32]. These are important proarrhythmic factors since they increase the substrate for ventricular arrhythmia development in the whole heart.

## 4. Remodeling of Repolarizing Transmembrane Currents in HF

The cellular mechanisms of prolonged repolarization are rather complex and it is generally accepted that it is the result of simultaneous downregulation and upregulation of various transmembrane ionic channels and/or transporters (Table 1).

**Figure 3:**
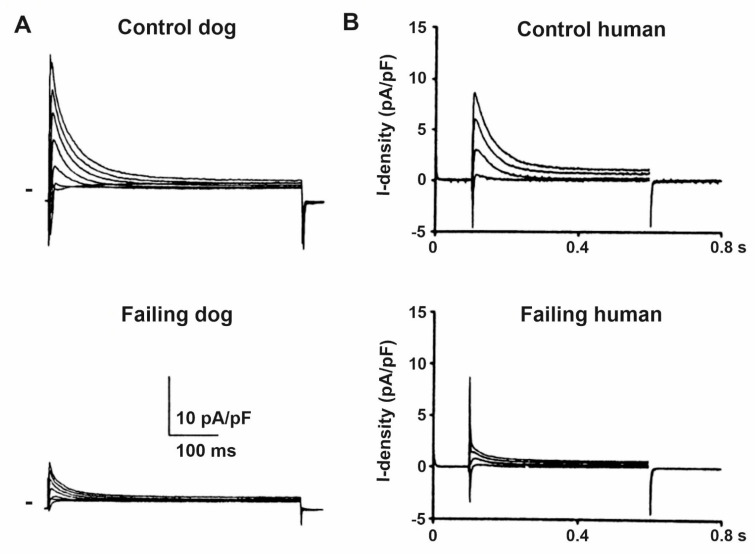
Representative original I_to_ recordings from (**A**) control and failing dog, and from (**B**) control and terminally failing human ventricular cardiomyocytes show significantly reduced I_to_ current density in heart failure. Zero current level is indicated by short bars on panel (**A**) [11,33].

The transient outward potassium current (I_to_) is the most frequently studied and established transmembrane current which exhibits downregulation (Figure 3) in HF [17,18,19,21,22,34]. Functional downregulation of I_to_ in HF was reported by several studies in humans [11,35]), dogs [17,19,36] and rabbits [20,34] which were associated with decreased expression of Kv4.3 mRNA [11,35,37] and protein [36] levels without changing or even enhancing those of Kv1.4 [18]. The Kv4.3 alpha channel subunit which is abundantly expressed in the dog [36,37] and human [38] considered to form fast I_to_ (I_tof_) that exhibits a fast (about 10–20 ms) recovery from inactivation [39,40] allowing it to operate in a wide range of heart rates. Slow I_to_ (I_tos_) recovers with a time course of seconds [39], it is expressed abundantly in rabbits [41] but relatively weakly in human ventricle conducted by Kv1.4 alpha channel subunits and its role in repolarization is not clear [42]. I_tof_ is responsible to form phase 1 repolarization and the notch afterward, influencing early repolarization and indirectly modulating the plateau voltage. In addition, an overlap of the steady-state activation and inactivation of I_to_ was also described in canine ventricular myocytes as I_to_ window current [40] contributing to phase 3 repolarization. Therefore, I_tof_ downregulation in HF can be expected to result in APD prolongation which is generally observed in HF. It is important to note that Kv4.3 channels exhibit two isoforms Kv4.3L and Kv4.3S [43] which are modulated differently by HF [18] in humans. The importance and possible role of I_tos_ changes in repolarization in HF is not clear since both its downregulation [37]) and upregulation [18] were described. Kv4.3 and Kv1.4 alpha channel proteins are associated with different accessory proteins such as KChiP2 and DPP6 and DPP10 which subunits can be also subject to downregulation in HF [34]. The abundant evidence supporting I_to_ downregulation in HF facilitated pharmacological research to develop drugs that enhance this current [44,45].

Downregulation of the slow (I_Ks_) and rapid (I_Kr_) delayed rectifier potassium currents were also reported in HF [12,17,19,21,22,34] which significantly contributes to repolarization prolongation or attenuation of repolarization reserve [46] in HF. The literature on changes of mRNA and protein expression of hERG Kv11.1 alpha subunits for I_Kr_ in HF is controversial, both decrease and no change were reported [34,35,47] on mRNA or protein levels raising the possibility of the importance of both post-transcriptional and post-translational modulation. Similar observations were published regarding LQT1 and MinK, MiRPs mRNA and proteins which make up ion channels for I_Ks_ [34,36,43]). In order to decrease the arrhythmogenic APD lengthening and enhanced dispersion of repolarization, activators of both I_Kr_ [48,49] and I_Ks_ [50] were developed and tested experimentally.

**Figure 4:**
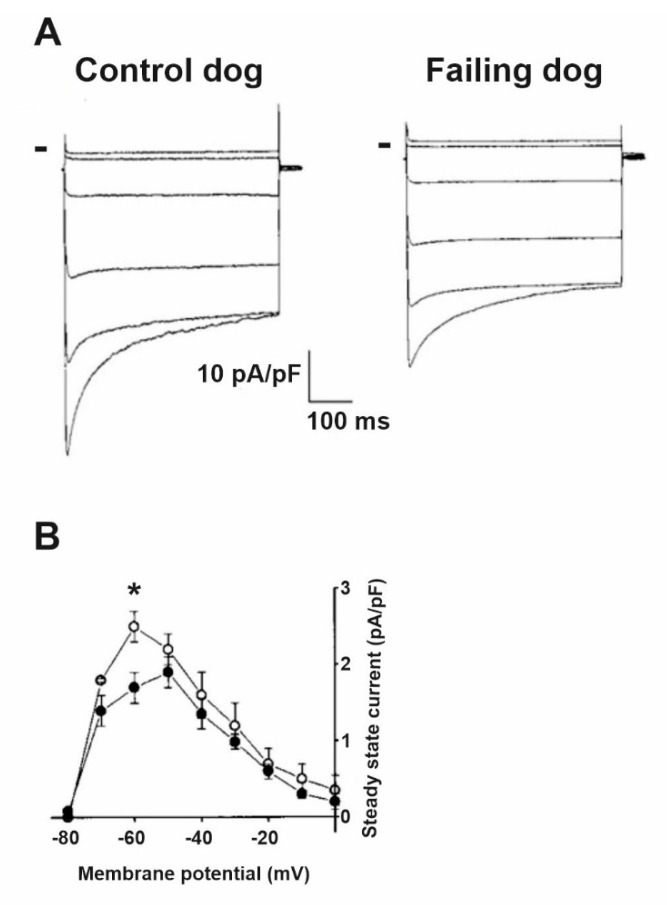
I_K1_ is downregulated in heart failure. (**A**) Representative current traces from control and failing dog ventricular myocytes using voltage steps between −150 mV and −50 mV using 20 mV increments. Peak and steady-state currents are reduced in failing myocytes. Small bars indicate zero current level. (**B**) The averaged steady-state current–voltage relation shows significantly reduced I_K1_ current density in failing myocytes (filled circles) compared to control (open circles) at −60 mV membrane potential [11]. * *p* < 0.05 vs control myocytes at −60 mV.

Decreased I_K1_ in HF was also reported (Figure 4) in rabbits [12,20], dogs [19] and humans [17]. Overexpression of CaMKII was shown in HF [51,52,53], and downregulation of I_K1_ was observed in mouse and rabbit myocytes with chronic CaMKIIδ_C_ overexpression [54]. It was recently described that miR1 can bind to Kir 2.1 channel proteins and decrease I_K1_ which can represent a so far unrecognized mechanism for channel modulation [55]. Recently, drugs enhancing I_K1_ were developed and tested in animal studies [56].

**Figure 5:**
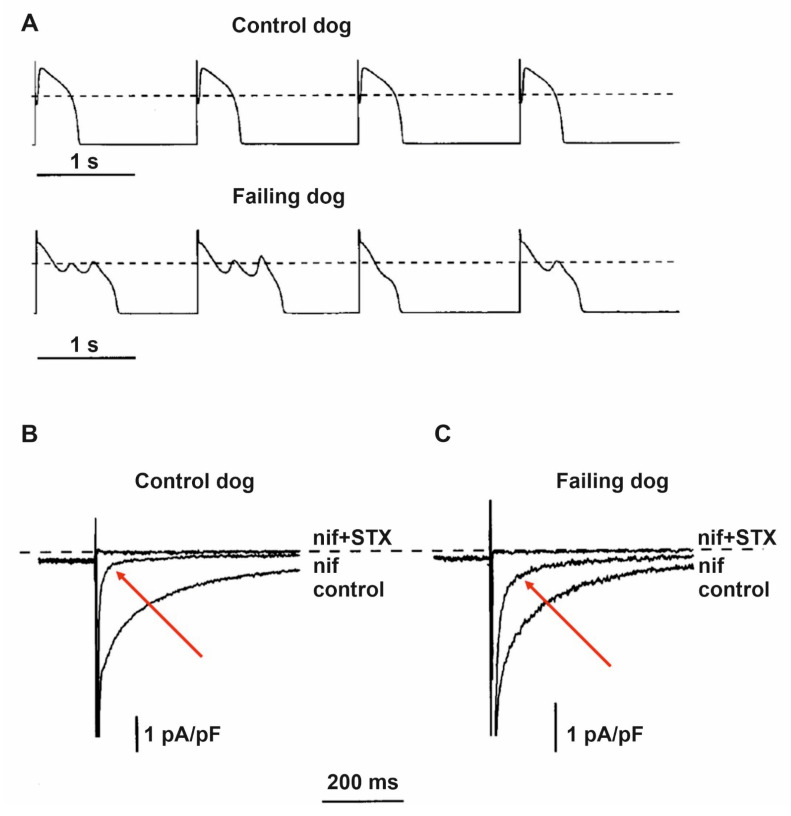
Increased late sodium current (I_NaL_) and repolarization abnormalities in dogs with heart failure. (**A**) Representative action potential recordings from ventricular myocytes isolated from normal and failing canine hearts. The action potentials in failing hearts exhibited prolonged duration, reduced notch, and early afterdepolarizations. Representative original inward current recordings from (**B**) normal and from (**C**) failing dog ventricular myocytes show significantly larger saxitoxin (STX; 1 μM) sensitive inward current following the application of the Ca^2+^ channel blocker nifedipine (indicated with red arrows; nif; 2 μM). The dashed line indicates zero current level [70].

In both dogs [57,58] and humans [26,59,60]), significantly slower decay of the inactivation of the inward sodium current (I_Na_) conducted by Nav1.5 channels was described in ventricular myocytes obtained from failing hearts, causing a sustained inward current, called late sodium current (I_NaL_) during the plateau phase of the action potential that also contributes to prolonged repolarization in HF (Figure 5). This augmented I_NaL_ facilitated by CaMKII [61,62,63] and may elicit significant APD prolongation resulting in arrhythmogenic early afterdepolarizations (EAD) and enhanced dispersion of repolarization [64]. It was also described that in canine failing cardiac ventricular myocytes the neuronal isoform Nav1.1 channels produce a larger contribution to the enhanced I_NaL_ than those of the cardiac Nav1.5 channels [58]. The increased I_NaL_, in addition to prolonging APD, also elevates intracellular Na^+^ concentration which in turn activates CaMKII and slows Ca^2+^ extrusion from the cells via the sodium–calcium exchanger (NCX) leading to increased sarcoplasmic (SR) Ca^2+^ content, and this can elicit delayed afterdepolarization (DAD) and consequent arrhythmias [65]. Based on these findings, specific inhibitors of I_NaL_ were developed and tested to treat HF-related arrhythmias [66,67,68,69].

Both increased function [20,71], and elevated expressions of mRNA [72] and proteins [73,74] of NCX were reported in HF. Therefore, in HF when both intracellular Ca^2+^ level and NCX function are augmented and since NCX extrudes one Ca^2+^ and transports three Na^+^ into the cell, a significant amount of surplus inward current can be generated to cause depolarizations that lead to triggered arrhythmias such as EAD and DAD [20]. This is further facilitated by the decreased outward current due to I_K1_ downregulation mentioned previously [12,17,20]. Therefore, NCX inhibitors were suggested for the management of arrhythmias in HF [75,76].

The small conductance calcium-activated, apamin-sensitive potassium current (SK2) was described originally in mouse atria [77] but was not verified in undiseased rat, canine and human ventricular tissue [78]. Recent studies, however, provided strong evidence that SK2 current or channel is upregulated in failing rabbit [79], dog [80] and human ventricles [81,82]. The pathophysiological role of SK2 channels in HF is not clear at present, since it may strengthen the impaired repolarization reserve and as such, it can have a protective antiarrhythmic influence [83]. Additionally, blocking SK2 channels by apamin led to the development of EADs and Torsades de Pointes arrhythmias [83]. On the other hand, apamin eliminated recurrent spontaneous ventricular fibrillation in failing rabbit hearts by eliminating increased sensitivity of SK2 channels to heterogeneous elevations of intracellular calcium caused by HF [84]. It was also reported that SK2 current in hypertrophied rat ventricle was upregulated by CaMKII [85]. Recently, inhibition of SK2 current in the submicromolar range was reported by ondansetron [86], and it was found that it increased vulnerability to ventricular fibrillation in a failing rabbit heart [87].

## 5. Impaired Impulse Conduction in Heart Failure

Impulse conduction is impaired in the failing heart which can be attributed to several factors. Recent findings emphasize the importance of the reduction of critical conduction velocity [88]. The fast sodium current (I_Na_) is the most important transmembrane ion current to elicit fast depolarization and consequent impulse propagation. Although an early study did not show differences between the amplitude of I_Na_ measured in human ventricular myocytes originating from healthy and failing hearts [89] later reports verified decreased I_Na_ in ventricular myocytes in various failing heart models compared to those of undiseased ones [90,91,92]. This was attributed to the diminished expression of Nav1.5 alpha channel subunits [91]. However, the fact that the conduction velocity slows down in a frequency-dependent manner, i.e., decreases at high frequencies, suggests that the function of the sodium channel is modified rather than its expression [93]. In a porcine ischemic HF model, CaMKII was found to be significantly upregulated [93], altering channel gating properties at rapid frequencies and reducing sodium current through phosphorylation of Nav1.5 [94]. The significant role of CaMKII in arrhythmogenesis is supported by the fact that overexpressed CaMKII increases the incidence of VF/VT [94], whereas the decrease in phosphorylated CaMKII levels induced by SERCA2a gene therapy prevented the frequency-dependent decrease in conduction velocity and was associated with arrhythmia suppression in MI pigs [93]. Thus, new treatment strategies based on the reduction of CaMKII levels may be promising antiarrhythmic therapies in the future. Electrical coupling between the myocytes is also a key factor that can influence impulse propagation [95]. Several reports indicate that coupling through the gap junctions is affected by HF [96,97,98,99,100,101,102]. Decreased expression of the most important gap junction protein, connexin43, was reported in the failing heart [96,97,98,99] and decreased gap junction function was also reported in HF or in calcium overloaded ventricular preparations [100,101,103]. However, Akar et al. [104] did not find a direct correlation between the expression level of Cx43 and impulse conduction velocity in tachypacing induced failing dog hearts, moreover, action potential upstroke differences were not observed in ventricular myocytes isolated from normal and failing hearts. Results show that the hypophosphorylated state and the redistribution of Cx43, i.e., its translocation from the intercalated disk to lateral cell borders, plays an important role in the decrease in impulse conduction velocity in the failing heart. In a recent report based on experiments after transaortic constriction induced HF in mice, the enhanced spontaneous calcium waves triggered extrasystoles due to a weakened current sink in poorly coupled myocytes [102].

**Figure 6:**
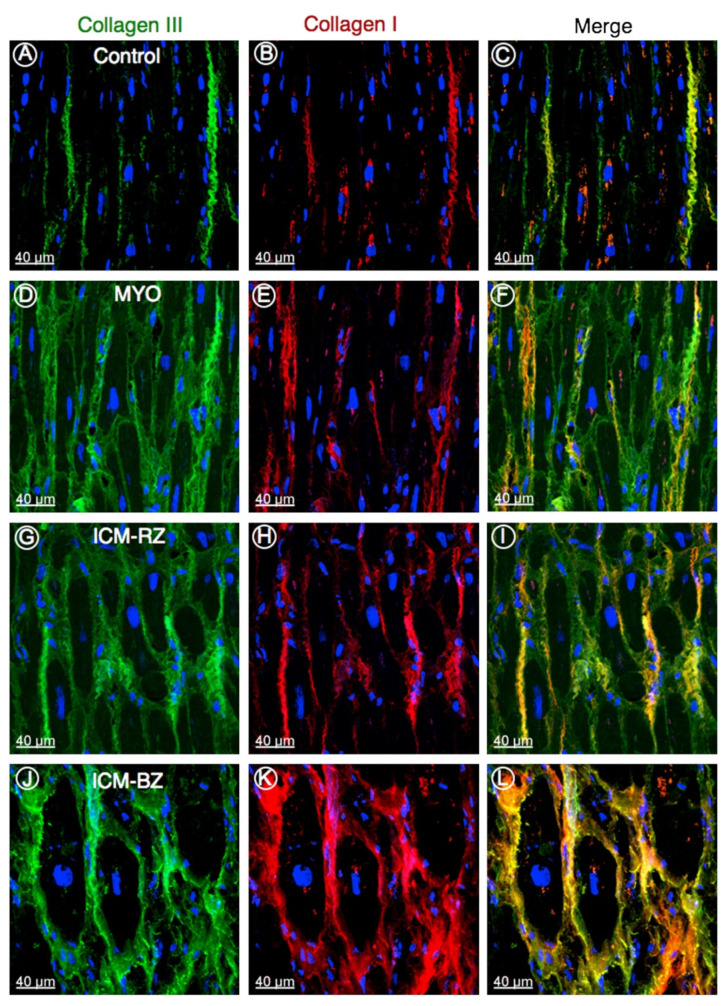
Representative confocal images of collagen type III (green) and type I (red) in control (**A**–**C**), in explanted human heart tissue samples from patients with inflammatory cardiomyopathy (MYO; **D**–**F**), from patients with ischemic cardiomyopathy (ICM) from the remote zone (ICM-RZ; **G**–**I**) and border zone regions (ICM-BZ; **J**–**L**), demonstrating an increased accumulation of fibrillar collagens in diseased myocardium. Nuclei are stained blue with 4′,6-diamidino-2-phenylindole [105].

Fibrosis (Figure 6) and scars are often observed in the failing heart [13,105]) which can produce anatomical barriers for impulse propagation and make conduction more heterogenous and are major contributors to the arrhythmia favoring reentrant arrhythmia (Figure 1A) development in HF [15]. Fibrotic alterations may impair impulse conduction and may also create conduction blocks. Remodeling of the border zone post-myocardial infarction alters the pattern and function of gap junctions, exacerbating heterogeneity of conduction [106]. In addition, increasing evidence highlights the role of the electrical interaction of myocytes and fibroblasts in arrhythmogenesis and conduction abnormalities [107]. Cardiac fibroblasts are electrically non-excitable cells that respond to mechanical deformation with a change in membrane potential via mechano-sensitive, non-selective cation channels [108,109,110,111,112,113,114]. Compression of the plasma membrane causes depolarization of the fibroblast membrane, while mechanical stretch hyperpolarizes the membrane [108,109]. Moreover, it was demonstrated that there is an electrical interaction between myocytes and fibroblasts via gap junctions [115,116,117,118], due to which all action potentials in myocytes induce characteristic membrane potential changes in fibroblasts [107]. On the other hand, Camelliti et al. found that electrical signals generated by mechanical stimulation in fibroblasts can be transmitted to myocytes [119]. Recent studies suggest that enhanced sensitivity of fibroblast membrane potential to mechanical effects in the diseased heart may effectively contribute to arrhythmogenic properties of the ischemic and failing heart [107].

## 6. Altered Transient Receptor Potential (TRP) Channels in Heart Failure

Transient Receptor Potential (TRP) channels are a group of nonselective cation channels that are sensitive to a wide spectrum of various environmental physical and chemical stimuli and contribute to intracellular calcium content alterations by increasing calcium influx [120,121,122,123,124]. The mammalian TRP superfamily can be grouped into six subfamilies according to their specific functions and sequence homology [125,126,127]: TRPC (Canonical), TRPV (Vanilloid), TRPM (Melastatin), TRPA (Ankyrin), TRPML (Mucolipin), and TRPP (Polycystic). The members of these subfamilies are similar in structure, containing six transmembrane helices [128]. Cation selectivity varies among members of the subfamilies: the channels are permeable to both monovalent and bivalent cations, most of them exhibit better Ca^2+^ than Na^+^ conductivity [129]. TRP channels are not voltage-gated channels but can be activated by various physical and chemical stimuli [130,131,132] such as mechanical stretch, fluctuations in temperature, intra, and extracellular ions and ligands (DAG, PIP2). A variety of vasoactive agents can stimulate TRP channels including endothelin-1, thrombin, ATP, angiotensin-II, or bradykinin [133,134,135,136].

The following TRP channels are expressed in the heart: TRPC, TRPV, TRPM, TRPA and TRPP. A total of seven members of TRPC channels are expressed on almost all cell types in the heart, and TRPC1, C3, C4, C5, and C6 are known to be overexpressed in heart failure [137,138]. TRPC channels play major roles in signal transduction in cardiac myocytes and some of them are activated by Ca^2+^ store depletion causing store-operated Ca^2+^ entry that may lead to proarrhythmic spontaneous Ca^2+^ waves [139], and this effect correlates well with the upregulation of TRPC3 and C4 in adult ventricular cardiomyocytes.

In addition, recent studies suggest that TRP channels are crucial in adverse cardiac remodeling, including cardiac hypertrophy and fibrosis, where roles for TRPCs, TRPVs and TRPMs were demonstrated, moreover, these channels are upregulated in heart failure [120,122,140]. The importance of TRPC channels is supported by the finding that TRPC3/C6 mice did not develop pressure-induced hypertrophy [141]. TRPC channels are of great importance in fibrosis also, which is supported by the observation that deletion of the TRPC3 gene did not result in fibrosis following angiotensin II infusion [142,143,144]. Compounds developed for the selective modulators of TRP channels may be of use for the prevention of adverse remodeling in the future, and may also have antiarrhythmic effects.

## 7. Alterations of Calcium Handling in Heart Failure

Heart failure alters calcium homeostasis in the ventricular muscle which includes transmembrane ion channels, receptors, transport proteins, intracellular mitochondria, calcium storage sites, signaling pathway enzymes and a number of the reported changes are still controversial and subject to debate [145,146,147]. To discuss all of these issues is far beyond the scope of this review and interested readers are encouraged to read comprehensive papers published on this topic [146,148,149,150,151,152]. In the present article, we only focus on processes that directly affect arrhythmogenesis.

**Figure 7:**
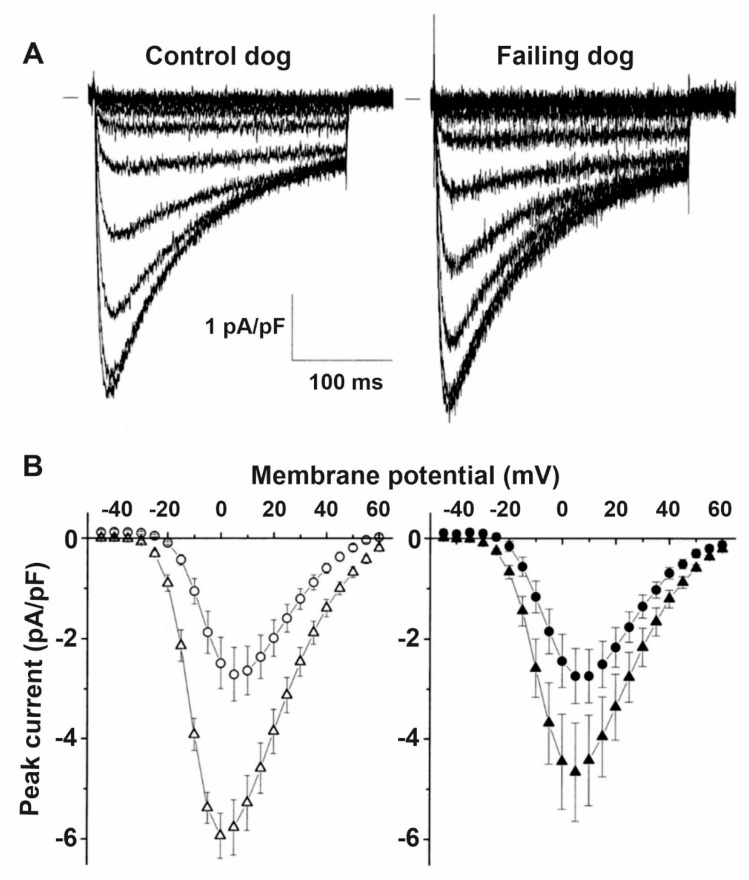
I_CaL_ is not altered by heart failure. (**A**) Representative family of Cd^2+^ sensitive difference currents measured from ventricular cardiomyocytes isolated from control and failing dog hearts, elicited by depolarizing voltage steps between −45 and +60 mV in 5 mV increments. Small bar indicates zero current level. (**B**) Average peak current–voltage relation of the Cd^2+^ sensitive difference currents in control and failing dog ventricular cardiomyocytes did not differ at baseline (open circles and filled circles, respectively). The stimulating effect of isoproterenol was attenuated in failing myocytes (filled triangles) compared to control myocytes (open triangles) [11].

**Figure 8:**
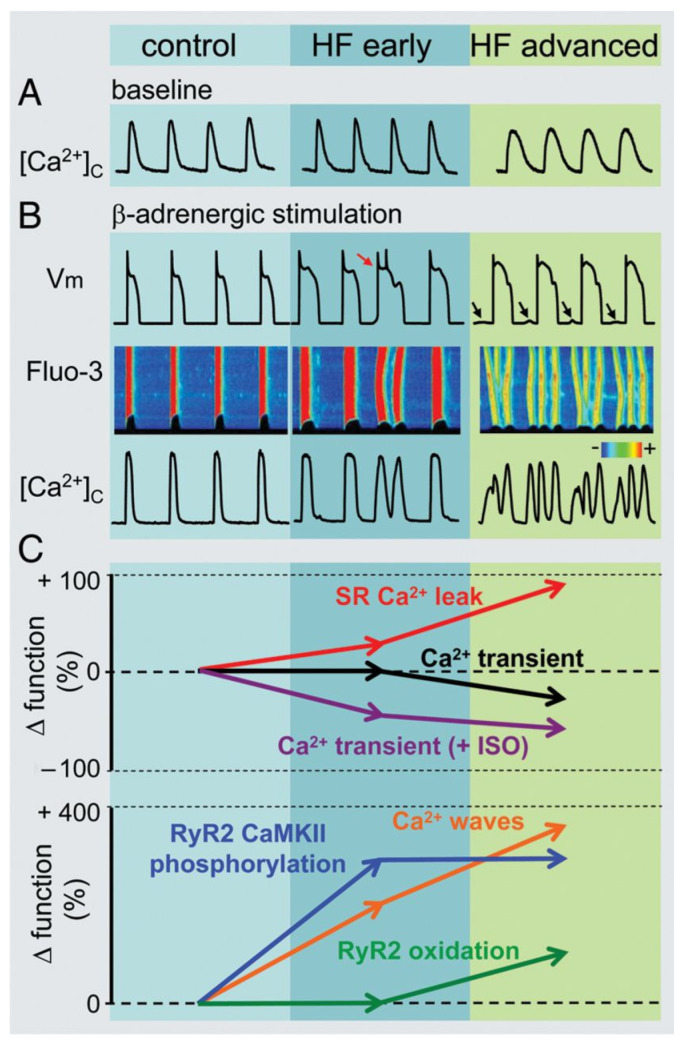
Heart failure progression and remodeling of calcium handling. (**A**) Representative Ca^2+^ transients recorded from dog isolated myocytes from controls, from early and advanced stages of heart failure. (**B**) Representative action potentials (Vm), along with line scan Fluo-3 fluorescence images and Ca^2+^ transients ([Ca^2+^]_c_) during β-adrenergic stimulation in control, early and advanced HF canine myocytes. The red arrow indicates an extrasystolic action potential. Delayed afterdepolarizations are indicated by arrows. (**C**) Summary graphs of altered Ca^2+^ handling in HF. Progression of HF causes early and progressive increase in sarcoplasmic reticulum Ca^2+^ leak (upper graph, red line). The amplitude of the baseline Ca^2+^ transient (upper graph, black line) decreases with significant time-delay compared to elevation of Ca^2+^ leak during the progression of HF. This is attributable to the capacity of myocyte Ca^2+^ handling for autoregulation. In contrast, the frequency of diastolic Ca^2+^ waves recorded following β-adrenergic stimulation by isoproterenol (ISO) (lower graph, orange line) parallels increases in the SR Ca^2+^ leak. Ca^2+^ waves facilitate diastolic SR Ca^2+^ loss leading to decreased in Ca^2+^ transient amplitude (+ISO; upper graph, purple line). Importantly, in advanced stages of HF, persistent cytosolic Ca^2+^ oscillations uncouple electrical excitation from mechanical response. Progressive alterations in Ca^2+^ handling in HF are coupled to sequential modification of RyR2 by CaMKII-dependent phosphorylation (lower graph, blue line) and oxidation (lower graph, green line) [161].

In the failing heart, the magnitude of the L-type calcium current (I_CaL_) is not changed (Figure 7) [153,154,155] but the decay of the calcium transient is slowed resulting in less calcium filling the SR and decreased systolic but enhanced diastolic cytosolic calcium levels [153,154,156]. These changes were associated with decreased ryanodine2 receptor (RyR2), SERCA2a and phospholamban protein expression [155]. The SR is leaky in the failing heart (Figure 8), spontaneously releasing Ca^2+^ into the cytosol [157,158] which is linked to increased phosphorylation of RyR2 [159,160]. As a consequence, the upregulated NCX [20,71] carries a significant amount of inward current in a situation where opposing outward currents, such as I_K1,_ are already diminished due to its downregulation [20]. These changes would facilitate the occurrence of DADs and consequent extrasystoles [12,20]. This latter enhanced ectopic activity could provide triggers for ventricular arrhythmias in situations where the arrhythmia substrate was already manifested by repolarization and conduction remodeling discussed earlier. In addition, enhanced NCX in the reverse mode can be proarrhythmic by contributing to calcium overload of the myocardium [75].

## 8. Changes of the Pacemaker Current (I_f_) in HF

**Figure 9:**
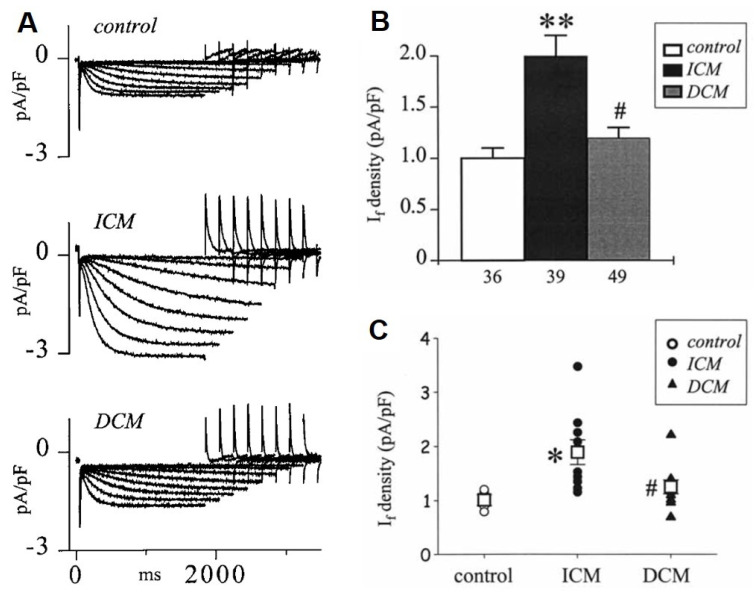
The I_f_ current is upregulated in human terminal heart failure due to ischemic (ICM) and dilated cardiomyopathy (DCM). (**A**) Representative original I_f_ current recordings from a control (donor heart), an explanted ICM and DCM heart, during membrane hyperpolarization to increasing negative potentials from −60 to −130 mV. (**B**) Summary graph of I_f_ density at −120 mV in control, ICM and DCM hearts; ** *p* < 0.001 vs. control, # *p* < 0.01 vs. ICM. (**C**) The average I_f_ densities measured in individual control, ICM and DCM hearts [165]; * *p* < 0.01 vs. control, # *p* < 0.05 vs. ICM. Open squares represent the corresponding mean for each group.

In the sinus node, evidence suggests dysfunction and downregulation of I_f_ in HF [9,10,162,163] which may cause bradyarrhythmia in certain situations. In contrast, in the ventricle, I_f_ is upregulated (Figure 9) [164,165,166] which was attributed to enhanced mRNA and protein expressions of HNC2 and HCN4 channels in the failing human ventricle [167]. The enhanced pacemaker current can induce extra beats leading to increased triggered activity for arrhythmias in the failing ventricle [168]. It was also reported recently that overexpression of HCN4 channels in mice did not change sinus node frequency but enhanced ventricular ectopic activity, disturbed calcium homeostasis and consequently increased arrhythmogenicity [169] (Table 1).

## 9. Changes in Cardiac Chloride Currents in the Failing Heart

So far, the genes encoding the following chloride channels have been identified in the heart: CFTR, ClC-2, ClC-3, CLCA, Bestrophin, and Ano1. These chloride channels are responsible for the following currents: the cystic fibrosis transmembrane conductance regulator (CFTR) for the protein kinase A (PKA), protein kinase C (PKC) and extracellular ATP activated Cl^−^ currents (I_Cl,PKA_, I_Cl,PKC_ and I_Cl,ATP_ respectively) [170,171,172,173,174,175,176,177]. ClC-2 is responsible for the inwardly rectifying Cl^−^ current (I_Cl,ir_) activated by hyperpolarization and cell swelling [178,179]. ClC-3 carries the volume-regulated outwardly rectifying Cl^−^ current (I_Cl,vol_), the basally activated (I_Cl,b_), and the swelling-activated (I_Cl,swell_) components [180,181,182,183,184,185,186,187,188]. Ano1 and CLCA-1 and Bestrophin underlie the Ca^2+^-activated Cl^−^ current (I_Cl,Ca_) [189,190,191,192,193,194,195]. A novel, extracellular acidosis activated Cl^−^ current (I_Cl,acid_) was also demonstrated in cardiac myocytes but the molecular identity for I_Cl,acid_ is currently not known. Recent transgenic studies have suggested that these chloride channels may play a significant role in arrhythmogenesis, hypertrophy and heart failure, as well as in cardioprotection following ischemia reperfusion [196].

The role of different channels in heart failure was investigated in several studies, the details of which go beyond the scope of this article. Changes in heart failure were described primarily in relation to CFTR, CIC-2, CIC-3 channels, and calcium-activated chloride current. However, the clinical and functional significance of these changes is unclear. In the case of CFTR, its downregulation and reversal of its endo-epicardial gradient, which may contribute to the increased risk of arrhythmia in patients with heart failure, were described. Recent studies have shown constitutive activation of I_Cl,swell_ in heart failure, however, its clinical significance is unclear. Calcium-activated chloride current can be an important mediator of apoptosis, however, information on the possible involvement in the pathogenesis of heart failure is also limited [197,198,199,200].

## 10. Atrial Remodeling in the Presence of Chronic Heart Failure

Heart failure is often associated with atrial fibrillation, and atrial fibrillation is the most common arrhythmia in heart failure [8] and the presence of AF represents an increased risk for all-cause mortality in HF patients [201] and a worse prognosis [202]. As the severity of chronic heart failure progresses, the prevalence of AF also increases [203,204]. The relatively frequent co-existence of AF and HF can be attributed to their shared risk factors (coronary artery disease, hypertension, valvular heart disease, etc.) and to their close pathophysiological relationship [205], including chronic elevated neurohumoral activity [206]. In addition, AF with a fast ventricular rate can result in cardiomyopathy and ventricular remodeling, and thus heart failure [207], while increased atrial pressure in HF increases atrial wall tension, induces atrial dilation and fibrosis, activates systemic neurohumoral pathways, and induces atrial remodeling promoting the development and maintenance of AF [208,209].

In animal models of HF, electrical, structural and Ca^2+^ homeostasis remodeling processes were identified in the atria that promote the development of AF, however, some of the elements of electrical and Ca^2+^ handling remodeling differ based on the model and duration of HF, the species used in experimental HF [210]. Interstitial fibrosis is the most prominent and reproducible (independent of species and model of HF used) change induced by HF in the atria [209,211,212,213,214]. Interestingly, the profibrotic remodeling response in HF seems to be more intense in the atria than in the ventricles [215] and significant alterations in different microRNAs were identified to underlie the atrial specific fibrotic response in an animal model of HF [216]. In a reversible canine model of tachypacing induced congestive heart failure, ionic remodeling but not the fibrotic changes showed complete reversal, suggesting that structural remodeling was the primary contributor to AF maintenance in experimental HF [217]. Recently it was also shown that HF increased profibrotic markers and caused calcium handling abnormalities in human atria and these abnormalities, including increased ryanodine-receptor open probability, serve as key triggers for AF initiation and cause further atrial electrical remodeling in patients with heart failure that promotes maintenance of the arrhythmia [218].

In a dog model of ventricular tachypacing induced HF, resting membrane potential and action potential amplitude in atrial myocytes was not altered in HF dogs [219]. The action potential duration (APD) was unchanged at 1 Hz baseline stimulating frequency, however, atrial myocytes isolated from HF dogs exhibited significantly longer APD at higher frequencies and this prolongation was larger as frequency increased [219]. The APD results obtained in isolated atrial cells paralleled prolonged effective refractory period findings in vivo in HF dogs [219]. These findings are also consistent with human data obtained from electro-anatomical mapping in HF patients [220]. However, it should be noted that relatively few and inconsistent data are available regarding atrial APD changes in patients with HF [221,222,223].

Atrial myocytes obtained from CHF dogs (heart failure induced by 6 weeks of ventricular tachypacing) exhibited decreased densities of several transmembrane currents [219]. The L-type Ca^2+^ current was reduced by ≈30%, I_to_ by ≈50% and the slow delayed rectifier K^+^ current was reduced by ≈30% while an approximate 45% increase in the transient inward Na^+^/Ca^2+^ exchanger current (I_NCX_) was observed. The I_K1_, ultrarapid and rapid delayed rectifier, and T-type Ca^2+^ currents were not influenced by CHF in this model. However, in another, more chronic (4 months of ventricular tachypacing) dog HF model, a different overall picture of atrial remodeling was described [224]. The APD of left atrial myocytes was shortened in parallel with right atrial ERP shortening, however, similar fibrotic changes were detected to those shown in the shorter duration HF dog model [224]. Increased I_to_, decreased I_Kur_, I_Ks_ and I_K1_ were reported, while I_CaL_ did not change [224]. The differing results regarding electrical remodeling in the two dog HF models suggest that there is a complex and time-dependent relationship between HF and the development of AF promoting atrial remodeling [225]. In addition, atrial electrical remodeling promoting AF markedly differs in the 6-week ventricular tachypacing induced canine CHF model from that observed in the canine atrial tachypacing induced AF model, where significant APD and ERP shortening, reduced I_CaL_ and I_to_, increased constitutive I_K,ACh_, unchanged I_K1_, I_Kr_, I_Ks_, I_Kur_ and I_CaT_ were observed [209,226,227,228,229,230]. In summary, time-dependent and different mechanisms seem to be responsible for AF promoting atrial remodeling in distinct animal models of HF, requiring careful extrapolation of the results in these models to human settings. While in atrial tachypacing induced atrial remodeling the wavelength decreases in a spatially heterogenous manner, thereby promoting reentry formation, the 6-week ventricular tachypacing induced CHF model involves atrial remodeling that increases the wavelength and promotes afterdepolarization-dependent atrial ectopic activity due to enhanced I_NCX_, triggering atrial fibrillation [209,219].

Disease-related AF promoting atrial remodeling was also observed in several clinical settings, with different ion channel expression profiles. In left ventricular dysfunction, increased atrial I_to_ was associated with atrial APD shortening with concomitantly unchanged I_CaL_ and I_K1_ [222]. However, in patients in sinus rhythm but with low ejection fraction and mitral valve disease, a significantly reduced I_CaL_ was found [231]. An earlier study also found decreased I_CaL_ in addition to reduced I_to_ in patients with atrial dilatation [232].

## 11. Need for Novel ECG Parameters for Improved Risk Stratification of Sudden Cardiac Death in HF

For risk stratification of SCD in patients with heart failure, the commonly used factor is decreased left ventricular ejection fraction (EF) [233]. However, EF has insufficient predictive value since a wide variability of annual SCD rates was observed in HF patients with reduced LV dysfunction [234,235] and has limited sensitivity since most SCD cases occur in patients with no significant left ventricular dysfunction [236]. Accordingly, several ECG parameters were also suggested to improve risk stratification for SCD in HF [237,238,239,240].

Although frequency corrected QT interval (QTc) prolongation is consistently found in HF patients [241,242], and in the MERLIN TIMI 36 trial, QTc prolongation was associated with an increased rate of SCD in patients with ischemic cardiomyopathy [243], growing evidence strongly suggests that lengthened repolarization alone does not directly translate to increased proarrhythmic risk [244,245,246,247,248]. Additional parameters characterizing repolarization were suggested as parameters for ventricular arrhythmia and/or SCD prediction.

The interval from the peak of the T wave to the end of the T wave (Tp-Te) on the 12-lead ECG reflects transmural dispersion of repolarization of the left ventricle [249]. Electrical remodeling is associated with an increase in transmural heterogeneity of repolarization, which predisposes the development of reentrant ventricular arrhythmias. Relatively few studies have examined the predictive value of Tp-Te in heart failure. However, Tp-Te was found to be predictive for VF and overall mortality in patients with LV dysfunction [250,251]. In addition, one study suggests that the Tp-Te value is a reliable predictor for VT and implantable cardioverter-defibrillator discharge in cardiac resynchronization therapy patients [252].

For the improved understanding of proarrhythmic repolarization disturbances caused by disease-related electrical remodeling and/or a wide array of cardiovascular and non-cardiovascular drugs, the concept of repolarization reserve was introduced [46,253,254,255]. Repolarization reserve highlights the redundant nature of cardiac repolarization capacity. In case the function of one repolarizing current is impaired or lost (e.g., due to current downregulation or inhibition by drugs), this does not always lead to large repolarization prolongation, since other currents can at least partially take over the lost function. However, the repolarizing capacity of the myocardium will be impaired making the heart more susceptible to arrhythmia development following even a relatively mild, additional repolarization inhibition. Based on the alterations of different repolarizing and depolarizing cardiac ionic currents in heart failure described in previous sections of this review, repolarization reserve is markedly impaired in patients with HF. One of the major repolarizing currents critically contributing to repolarization reserve [256] is consistently reported to be downregulated as part of the electrical remodeling in HF [12,17,19,21,22,34]. In addition, I_to_ is downregulated in experimental models of HF and in patients with HF, further impairing repolarization reserve [11,17,19,20,34,36]. As mentioned above, impaired repolarization reserve does not lead to marked QT prolongation on the ECG and QT prolongation *per se* does not predict proarrhythmic risk adequately, therefore, beat-to-beat variability of repolarization, characterizing temporal lability of repolarization and quantified as the short-term variability of the QT interval (STVQT) was suggested as a novel surrogate biomarker with improved predictive value for severe ventricular arrhythmias [239,257,258]. Indeed, in animal experimental studies [246,259,260,261,262] and in several patient populations (including HF) with increased susceptibility for ventricular arrhythmias and with impaired repolarization reserve, STVQT was significantly increased and correlated better with ventricular arrhythmia development than QT prolongation [263,264,265,266,267], making STVQT a promising proarrhythmia marker.

## 12. Summary

Ventricular arrhythmias and atrial fibrillation commonly occur and are key contributors to morbidity and mortality in patients with heart failure. Heart failure patients exhibit increased susceptibility to cardiac arrhythmias. Approximately one-third to almost half of HF patients are lost due to SCD caused by lethal ventricular arrhythmias. Atrial fibrillation and HF are frequently diagnosed together. Atrial fibrillation can lead to HF and AF often develops in patients with HF, their coexistence significantly worsens the prognosis. Disease-related electrical and structural remodeling occurs in ventricular and atrial tissue in HF that promote the development and maintenance of ventricular and atrial arrhythmias. Remodeling increases the arrhythmia substrate: marked fibrotic alterations, scar development, reduced peak sodium current and altered connexin expression impair impulse conduction and increase impulse propagation heterogeneity both in the atria and ventricles in HF. Significant prolongation of repolarization and increased dispersion of repolarization, caused by downregulation of several potassium currents and upregulation of the late sodium current, further enhance the arrhythmia substrate in HF. Impaired conduction and heterogenous repolarization create the substrate for reentry arrhythmias. Arrhythmogenic triggers are provided by upregulation of I_f_ and NCX in the ventricles as well as pathologically altered calcium handling, involving abnormal SR and CaMKII function, leading to early and delayed afterdepolarization development. The identification of key elements of the molecular background of arrhythmogenic electrical and structural remodeling enables future efforts to develop novel treatment modalities for more efficacious arrhythmia management in the setting of heart failure and atrial fibrillation.

## Figures and Tables

**Table 1:** Remodeling of transmembrane ionic currents in failing ventricular muscle.

Current	Change	References
I_Na_ peak	**↓**	[25]
I_NaL_	**↑**	[61,64,70]
I_CaL_	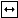	[11,154,155]
I_to_	**↓**	[21,34,35,36]
I_K1_	**↓**	[12,17,19]
I_Kr_	**↓**	[12,19,34]
I_Ks_	**↓**	[12,17,19,21]
I_KCa_ (SK2)	**↑**	[79,80,81,82]
I_f_	**↑**	[164,165,167]
I_NCX_	**↑**	[20,71]
Connexin	**↓**	[96,160]

I_CaL_, L-type Ca^2+^ current; I_f_, funny/pacemaker current; I_KCa_, calcium-activated potassium current; I_Kr_, rapid component of delayed rectifier potassium current; I_Ks_, slow component of delayed rectifier potassium current; I_K1_, inward rectifier potassium current; I_Na_, sodium current; I_NaL_, late sodium current; I_to_, transient outward current; I_NCX_, Na^+^/Ca^2+^ exchanger current.
